# “Hit-and-Run” transcription: *de novo* transcription initiated by a transient bZIP1 “hit” persists after the “run”

**DOI:** 10.1186/s12864-016-2410-2

**Published:** 2016-02-03

**Authors:** Joan Doidy, Ying Li, Benjamin Neymotin, Molly B. Edwards, Kranthi Varala, David Gresham, Gloria M. Coruzzi

**Affiliations:** Center for Genomics and Systems Biology, Department of Biology, New York University, New York, NY 10003 USA

**Keywords:** Transcription factor (TF), Target gene, 4-thiouracil (4tU), Dynamic regulation, Gene regulatory networks, “hit-and-run” transcription, Transcriptional regulation

## Abstract

**Background:**

Dynamic transcriptional regulation is critical for an organism’s response to environmental signals and yet remains elusive to capture. Such transcriptional regulation is mediated by master transcription factors (TF) that control large gene regulatory networks. Recently, we described a dynamic mode of TF regulation named “hit-and-run”. This model proposes that master TF can interact transiently with a set of targets, but the transcription of these transient targets continues after the TF dissociation from the target promoter. However, experimental evidence validating active transcription of the transient TF-targets is still lacking.

**Results:**

Here, we show that active transcription continues after transient TF-target interactions by tracking *de novo* synthesis of RNAs made in response to TF nuclear import. To do this, we introduced an affinity-labeled 4-thiouracil (4tU) nucleobase to specifically isolate newly synthesized transcripts following conditional TF nuclear import. Thus, we extended the *TARGET* system (Transient Assay Reporting Genome-wide Effects of Transcription factors) to include 4tU-labeling and named this new technology *TARGET*-*tU*. Our proof-of-principle example is the master TF Basic Leucine Zipper 1 (bZIP1), a central integrator of metabolic signaling in plants. Using *TARGET*-*tU*, we captured newly synthesized mRNAs made in response to bZIP1 nuclear import at a time when bZIP1 is no longer detectably bound to its target. Thus, the analysis of *de novo* transcripomics demonstrates that bZIP1 may act as a catalyst TF to initiate a transcriptional complex (“hit”), after which active transcription by RNA polymerase continues without the TF being bound to the gene promoter (“run”).

**Conclusion:**

Our findings provide experimental proof for active transcription of transient TF-targets supporting a “hit-and-run” mode of action. This dynamic regulatory model allows a master TF to catalytically propagate rapid and broad transcriptional responses to changes in environment. Thus, the functional read-out of *de novo* transcripts produced by transient TF-target interactions allowed us to capture new models for genome-wide transcriptional control.

**Electronic supplementary material:**

The online version of this article (doi:10.1186/s12864-016-2410-2) contains supplementary material, which is available to authorized users.

## Background

The ability of plants to mount rapid responses to changes in nutrient signals is key to their survival. In response to extracellular signals, a cell must regulate the expression of thousands of genes within a short time. For example, plant roots mount a broad transcriptional response within just three minutes of exposure to external nitrate treatment [1]. In this process, the signals are transduced by master regulator transcription factors (TFs), initiating the expression of their target genes and propagating rapid and broad transcriptional responses to environmental changes [2, 3]. However, capturing and modeling the dynamics of such rapid signal-induced responses in gene regulatory networks remains challenging [4, 5].

Recently, a dynamic mode of TF regulation named “hit-and-run” was described for the master regulator Basic Leucine Zipper 1 (bZIP1) in propagating nitrogen nutrient signals [6]. In this “hit-and-run” model, a master TF binds to the promoter of a set of “transient” gene targets (the “hit”) to initiate transcription, and then leaves the promoters (the “run”), while the transcription of the “transient” targets continues even after the TF dissociation. This “hit-and-run” model of continued transcription resulting from a transient interaction with a master TF was first proposed decades ago [7]. This “hit-and-run” model has gained experimental support now in the genomic era, and has been reinvoked to enable rapid activation of a network of genes in response to nutrient signals [4, 6, 8]. However, the prior studies measured levels of steady state mRNA – and not nascent transcripts– thus, irrevocable evidence supporting the continued *de novo* transcription of “transient” targets after TF dissociation is still lacking.

**Fig. 1:**
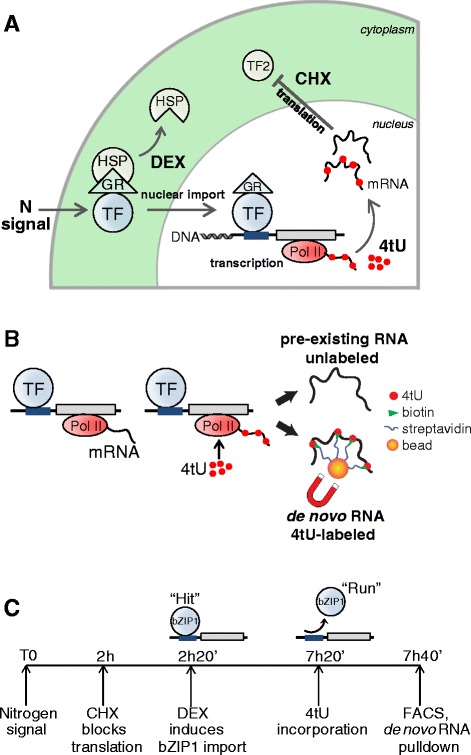
*TARGET*-*tU* identifies actively transcribed TF targets. Schematic of the *TARGET*-*tU* system. **a** Protoplasts (plant cells dissociated from whole roots) transfected with a 35S::GR::TF construct are sequentially treated with: i) the nitrogen (N) signal transduced by the TF, ii) cycloheximide (CHX) to block translation, allowing RNA synthesis of only primary TF targets, iii) dexamethasone (DEX) to release the GR-TF fusion from the cytoplasmic heat shock complex (HSP), inducing nuclear import. Five hours after DEX-induction of TF nuclear localization, cells were exposed to iv) 4-thiouracil (4tU) so that thio-labeled UTP nucleotides are incorporated into newly synthesized RNA (see also **c** and Additional file 2: Figure S1). **b** Thiol-specific biotinylation and pull-down with streptavidin-coated magnetic beads enable selection of newly synthesized transcripts apart from pre-existing transcripts. **c** Timeline of the sequential treatments described in this study. Cell protoplasts were exposed to 4tU nucleobase 5 h after bZIP1 nuclear activation, to show the continued transcription of “hit-and run” targets

Here, we used a novel experimental approach to capture nascent transcripts by assaying *de novo* synthesis of mRNAs in response to conditional import of a TF into the nucleus (Fig. 1). Standard transcriptional assays measure total cellular levels of mRNA. In these assays, changes in mRNA levels of target genes in response to TF perturbation cannot be quantifiably attributed to *de novo* RNA synthesis at the time of assaying. Thus, we developed an approach to track *de novo* RNA synthesis in response to TF nuclear import.

The introduction of a nucleobase analogue, 4-thiouracil (4tU), allows affinity-based capture of *de novo* synthesized RNA [9, 10]. When cells or organisms are exposed to 4tU, RNA synthesized post-introduction will incorporate thio-substituted UTP nucleotides into their sequence. This approach represents the state-of-the-art methodology to study transcription dynamics in model organisms [11–13], and was recently adapted in Arabidopsis to determine transcription rates in response to changes in temperature [14]. In our current study, we developed a new application of this approach by combining TF-perturbation with 4tU-labeling, to capture newly synthesized transcripts of dynamic TF target interactions, including ones resulting from transient bZIP1-target binding. Using this system, we detected the continued generation of new transcripts after transient TF-target binding and dissociation of bZIP1 from the promoter of its gene targets. These results provide clear and direct evidence of sustained transcription of transient targets beyond TF dissociation and thus support the “hit-and-run” model of transcription.

## Results and discussion

### Combining conditional activation of TF with 4tU-labeling to capture *de novo* transcribed targets

We modified the cell-based TF perturbation assay called *TARGET* (Transient Assay Reporting Genome-wide Effects of Transcription factors), which can identify primary TF targets from either TF-regulation (by transcriptomics) or TF-binding (by ChIP-Seq) events assayed in the same cell samples [6, 15]. Herein, we extended the *TARGET* system to include 4tU-labeling (*TARGET*-*tU* pronounced *TARGET* “*two*”), which enabled us to capture *de novo* RNA synthesis induced by the conditional nuclear import of a TF-of-interest (Fig. 1a). *TARGET* and *TARGET*-*tU* are comparable with the main modifications applied in *TARGET*-*tU* being the introduction of 4-thiouracil (Additional file 1: Table S1). In the *TARGET*-*tU* assay, the TF-of-interest is expressed in isolated root cells, but is retained in the cytoplasm due to the interaction between the fused glucocorticoid receptor (GR) tag and the cytoplasmic heat shock protein (HSP90). Treatment with dexamethasone (DEX) disrupts the GR-HSP90 complex, allowing nuclear import of the TF. This conditional nuclear localization of the TF in the presence of 4tU enables the incorporation of labeled UTP into actively transcribed TF-targets (Fig. 1a). By performing DEX-induction of nuclear import following a pretreatment with cycloheximide (CHX, Fig. 1c), we can identify direct targets of a TF in the *TARGET* system [6, 15, 16], as has also been shown previously in whole plants [17].

One major advantage of 4tU-tagging of mRNA is that it covalently labels nascent transcripts only, and therefore it is ideally suited for detecting dynamic changes in transcription of transient TF-target interactions. Using affinity capture, nascent 4tU-labeled RNA can be distinguished from pre-existing unlabeled RNA (Fig. 1b). Conditional induction of TF nuclear import combined with metabolic 4tU-labeling of nascent transcripts, to our knowledge, has not previously been used in any other organisms. Importantly, the *TARGET*-*tU* approach can be adapted to study any candidate TF, providing a robust means of identifying actively transcribed TF-targets in the context of dynamic gene regulatory networks.

### Capturing actively transcribed bZIP1 targets

**Fig. 2:**
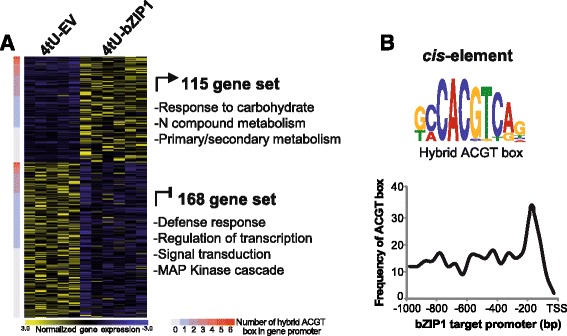
Actively transcribed targets of the master TF regulator bZIP1. **a** Transcriptomics profile of 4tU-labeled mRNA fractions from cells overexpressing bZIP1 (4tU-bZIP1) and empty vector control (4tU-EV), 5 h after bZIP1 activation. A heat map shows the expression profiles of 115 genes induced and 168 genes repressed by bZIP1 using 4tU-labeling and selection of actively transcribed genes. Heat map shows z-score normalized expression for genes/rows (from light yellow to dark blue gradient) using Mev [29]. Actively induced and repressed bZIP1 targets are ranked according to the number of ACGT hybrid box (0–6 boxes: from blue to red gradient) present in the 1 kb promoter regions upstream of the transcription start site (TSS). Gene ontology (GO) terms over-representation of actively induced and repressed gene sets selected from a singular enrichment analysis using Arabidopsis genome (TAIR10) as a reference with an FDR cutoff of 5 % (also see Additional file 4: Figure S2 for complete GO terms enrichment). **b** Identification of the known bZIP1 ACGT binding motif based on *cis*-elements discovery using MEME [32] (top panel, 45 sites, e-value = 6.2e^−11^) and known *cis*-element enrichment using Elefinder [33] (bottom panel, 299 sites, e-value = 8.2e^−5^) using 1 kb promoter regions. The frequency of ACGT hybrid box identified by Elefinder in the 1 kb promoter regions of bZIP1 targets were plotted to show the proximity of binding sites relative to the canonical transcription start site

We applied the *TARGET*-*tU* approach to study the mode of action of a master TF Basic Leucine Zipper 1 (bZIP1), a central integrator of metabolic signaling by carbon and nitrogen in plants [6, 18–22]. Specifically, to identify actively transcribed direct bZIP1 targets, following conditional TF nuclear import, we compared 4tU-labeled fractions between bZIP1 expressing cells (4tU-bZIP1) and an empty vector control (4tU-EV, Additional file 2: Figure S1) using microarrays. This enabled us to identify 283 newly synthesized mRNAs in the bZIP1-transfected cells, compared to the empty vector control (Fig. 2a). These direct bZIP1 targets shown in the heatmap in Fig. 2a, correspond to 115 genes whose transcription is actively induced by bZIP1, and 168 genes whose transcription is repressed by bZIP1 at the time of assaying (Additional file 3: Dataset S1).

These 283 genes whose transcription is initiated or repressed by bZIP1 nuclear import are significantly enriched for gene ontology (GO) terms associated with the known functions of bZIP1 including, regulation of transcription as well as primary and secondary metabolic processes (Additional file 4: Figure S2; [21]). In addition, *cis*-element analysis of the promoter regions of these target genes identified the known bZIP1-binding site (Fig. 2b; [23]). The bZIP-binding site (AGCT) was found in 170 bZIP1 targets (77 induced and 93 repressed direct targets), possessing up to 6 ACGT binding boxes in their promoters (Fig. 2a, Additional file 3: Dataset S1). This represents a total of 299 ACGT binding boxes identified in target genes specifically enriched in the proximal regions of the promoters (Fig. 2b), confirming that bZIP1 binds most proximal to the transcription start site [2]. Altogether, these results are consistent with actively transcribed genes captured by 4tU-labeling being direct targets of bZIP1.

### Comparison of *de novo* transcribed targets from 4tU-labeled RNA to targets from total RNA

**Fig. 3:**
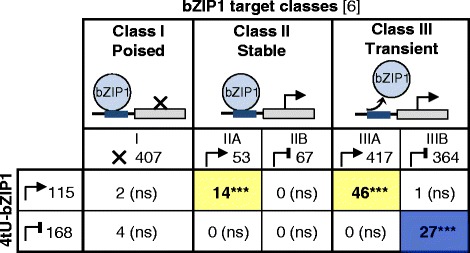
Active transcription persists 5 h after TF-induced nuclear entry, in both stably- and transiently-bound bZIP1 targets. A comparison of the overlap between actively transcribed bZIP1 targets captured from 4tU-labeled fractions (this study), and the previously reported classes of bZIP1 targets identified from total RNA [6]. Significant overlaps of induced genes (Class IIA & IIIA) and bZIP1-repressed genes (Class IIB & IIIB) are highlighted in yellow and blue, respectively. Intersects were performed using the “Genesect” function in VirtualPlant [30]. The significance of the overlaps from bZIP1 targets captured from 4tU-labeled fractions were verified using a hypergeometric test with Class IIA (stably-bound bZIP1 induced, *p* = 2.2e^−22^), Class IIIA (transiently-bound bZIP1 induced, *p* = 6.8e^−50^) and Class IIIB (transiently-bound bZIP1 repressed, *p* = 2.2e^−20^) targets

**Fig. 4:**
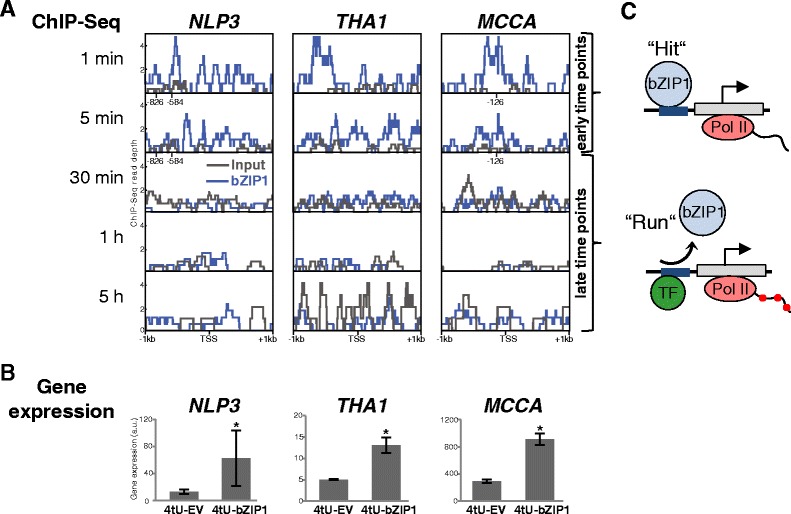
Transient targets initiated by a bZIP1 “hit” are actively transcribed after the TF has “run”. **a**-**b** bZIP1 mediates rapid and catalytic transcription in response to nitrogen signal. **a** Time-series ChIP-Seq binding 1 kb upstream and downstream of the transcription start site (TSS) for bZIP1 transient targets, *NLP3*, *THA1* and *MCCA* at 1 and 5 min (early time-points), 30 min, 1 and 5 h (late time-points) after induced nuclear localization of bZIP1. The bZIP-binding motifs identified by known motif enrichment (also see Fig. 2) were placed on the x-axis of early time-points (2 ACGT boxes in *NLP3* promoter at −826 and -584 bp, and 1 box in *MCCA* promoter at -126 bp). The bZIP1 ChIP-Seq time-course analyzed in this study was initially performed in [6]. **b** Gene expression levels of transient bZIP1 targets *NLP3*, *THA1* and *MCCA* in 4tU-labeled fractions of bZIP1 expressing cells (4tU-bZIP1) compared to 4tU-labeled fractions of empty vector (4tU-EV; *FDR < 0.1). Please note that the ChIP-Seq time course (**a**) and *de novo* transcriptomics (**b**) data were performed in two independent experiments. **c** “Hit and Run” mode of transcription for transient TF-targets

The differentially transcribed targets identified from 4tU-labeled RNA fractions were then compared to previously reported bZIP1 targets identified from total mRNA (Fig. 3; [6]). In the previous study, bZIP1 was proposed to mediate metabolic signals through a “hit-and-run” model of transcription. Based on analysis of direct targets identified by TF-regulation (microarray) or TF-binding (ChIP-Seq), Para *et al*., [6] uncovered three different classes of direct bZIP1 targets: Class I “poised” targets (TF bound but target not regulated), Class II “stable” targets (TF bound and gene regulation), and the largest Class III “transient” targets (gene regulation without observable TF-binding). In our prior *TARGET* study, regulation of target genes was determined as changes in steady-state mRNA at 5 h after bZIP1 nuclear import [6]. In this current study, we started the incorporation of the 4tU-labeled nucleobase 5 h after bZIP1 nuclear activation, for an additional 20 min (Fig. 1c, Additional file 2: Figure S1). Time-course ChIP-Seq studies showed that at this late time-point, bZIP1 has “run”, and the TF is no longer bound to the promoter region of its Class III transient targets (Fig. 3, Fig. 4a; [6]). In our present study, the functional read-out captured by 4tU-labeling enables us to determine if such transient bZIP1 targets are indeed *de novo* transcribed at times when the binding analysis shows that bZIP1 is no longer bound to the promoters.

Overall, we find significant overlaps between *de novo* transcribed targets captured from 4tU-labeled RNA, and known bZIP1 targets identified from total mRNA studies [6] both performed at 5 h after bZIP1 nuclear transport (Fig. 3). Further, we observed large and highly significant overlaps between actively transcribed targets identified using *TARGET*-*tU*, and previously reported bZIP1 targets including stably-bound bZIP1 targets (Class II) and transiently-bound bZIP1 targets (Class III) (Fig. 3, Additional file 3: Dataset S1). Over 50 % of the bZIP1 *de novo* induced targets (60 out of 115 genes), were previously identified as bZIP1 induced primary targets based on steady state mRNA [6] (Fig. 3). However, the overlap of the current 4tU studies with genes repressed by bZIP1 based on steady state mRNA [6], captured a lower number of repressed bZIP1 targets, specifically within the Class II “stable” targets. These results indicate that the new *TARGET*-*tU* and the original *TARGET* methods [6, 15] are more robust to capture induced targets, than repressed targets. By contrast, for bZIP1 repressed targets, down-regulation in mRNA level can be caused by reduced transcription rate, but also by active mRNA degradation (e.g. through inherent instability or through miRNA directed cleavage). Nevertheless, *TARGET*-*tU* captured a large number of repressed bZIP1 targets (Fig. 2a) that have been overlooked by our prior bZIP1 *TARGET* studies based on steady state mRNA [6] (Fig. 3). Thus, the *TARGET*-*tU* approach enabled us to successfully recover a highly significant number of both up- and down-regulated targets (Fig. 3; Class IIA (stable, induced) = 14 genes (*p* = 2.2e^−22^); Class IIIA (transient, induced) = 46 genes (*p* = 6.8e^−50^); Class IIIB (transient, repressed) = 27 genes (*p* = 2.2e^−20^), confirming that bZIP1 is a dual-mode regulator (activator and repressor). Also consistent with previous findings, Class I “poised” bZIP1 targets, to which bZIP1 is bound but are not regulated [6], were not identified in the 4tU fractions of actively transcribed bZIP1 targets. Altogether, these results confirm that we successfully captured actively transcribed TF-targets, which are a significant subset of the bZIP1 target genes identified by steady-state mRNA studies.

Here, we captured a subset of 73 transient targets identified from the original *TARGET* approach based on steady-state mRNA [6] and from our new *TARGET*-*tU* study which captures only actively transcribed targets, 5 h after bZIP1 nuclear import (Fig. 3). While this overlap between the *TARGET* and *TARGET*-*tU* experiments is significant, the numbers are low. Although both methods *TARGET* [6, 15] and *TARGET*-*tU* (this study) are performed in comparable experimental setups (Additional file 1: Table S1), they likely assay different profiles of mRNA targets (Additional file 5: Figure S3). Indeed, the original *TARGET* [6, 15] measures the steady state pools of mRNA targets accumulated during the 5 h of TF activation. By contrast, *TARGET*-*tU* only captures actively transcribed targets after introduction of labeled nucleobases (Additional file 5: Figure S3). Thus, *TARGET*-*tU* is able to distinguish whether a gene is actively transcribed without hinderance of detection above background levels of pre-existing mRNA. This may explain the large number of novel bZIP1 targets detected using *TARGET*-*tU*, especially for the targets that are actively repressed by bZIP1 (Fig. 2a), and the significant - yet partial - overlaps between *TARGET* and *TARGET*-*tU* (Fig. 3). Nevertheless, combining both approaches, we captured a subset of 73 dynamic bZIP1 targets with very high significance and therefore validated the “hit-and-run” mode of action for this master TF.

### Capturing *de novo* transcription proves that “hit-and-run” transcription persists beyond TF dissociation

The *TARGET*-*tU* approach, which identifies transient targets whose transcription is bZIP1 dependent, shows that such targets are actively transcribed when bZIP1 is no longer bound. Importantly, these transient targets include bZIP1 targets previously associated with nitrogen signaling (Fig. 4a and b). Indeed, the transiently-bound bZIP1 targets include *NLP3* (*NIN*-*LIKE PROTEIN 3*), which belongs to an important TF family for early response to nitrate signaling in Arabidopsis [24]. Other transient bZIP1 targets validated by our 4tU study include *THA1* (*THREONINE ALDOLASE 1*) and *MCCA* (*3*-*METHYLCROTONYL*-*COA CARBOXYLASE*), which encode enzymes involved in the catabolism of amino acids [25, 26]. Time-series ChIP-Seq assays show that bZIP1 binds to the promoter of these transient bZIP1 targets (*NLP3*, *THA1* and *MCCA*) within 1 and 5 min of the TF nuclear entry (Fig. 4a). We also confirm that ChIP-Seq signals identified in *NLP3* and *MCCA* promoters at early time-points, coincide with the presence of bZIP-binding motifs (Fig. 4a). However, at late time-points (30 min, 1 h and 5 h), the bZIP1 ChIP-seq signal peaks (Fig. 4a, blue peaks) are not significantly higher to the DNA input control (Fig. 4a, grey peaks). Therefore, while bZIP1 is bound to the transient targets at 1–5 min, it is no longer bound to the promoters of *NLP3*, *THA1* and *MCCA at* 30, 60 min or 5 h after bZIP1 nuclear import (Fig. 4a; [6]). Interestingly, active transcription of these transient targets continues after bZIP1 has “run”, as shown by transcriptomics of 4tU-labeled fractions (Fig. 4b).

Since the results from the ChIP-Seq time course (Fig. 4a; [6]) and *de novo* transcriptomics (Fig. 4b) were analyzed from two different studies, we performed a second replication of the ChIP-Seq experiments to confirm the “hit-and-run” model of transcription. This replication of the bZIP1 ChIP-Seq experiment was performed at the 5 h time-point (the same time-point as the *TARGET*-*tU*). First, we compared this new replicate to the previous binding data from Para et al. [6] and confirmed that we obtain the same bZIP1 targets (70 % overlap, pval < 1e^−10^) from the two independent ChIP-Seq experiments (Additional file 6: Figure S4A). We also confirm that bZIP1 is no longer bound to the promoter of the transient targets 5 h after its nuclear entry, using two independent experiments (Additional file 6: Figure S4B, D). To further support the “hit-and-run” model, we show 10 additional examples of transient targets captured in *de novo* transcribed fractions using the *TARGET*-*tU* approach and we juxtaposed their binding and *TARGET*-*tU* expression profiles (Additional file 6: Figure S4B-E), confirming the active transcription of these dynamic bZIP1 targets.

Thus, our findings provide experimental support for a “hit-and-run” transcription model, which posits that transcription is initiated when the TF “hits” the gene promoter to organize a transcriptional complex, after which transcription by RNA polymerase continues after the TF “runs”. In this model, we postulate that master signal transducers like bZIP1 may act as “catalyst TFs”, possibly by physical recruitment of other TF partners (Fig. 4c). We also propose that such dynamic mode of action rapidly activates large sets of genes in response to environmental changes.

## Conclusion

Here, the novelty of our system is the temporal TF-activation capturing primary targets combined with 4tU-labeling capturing *de novo* transcriptome. Thus, our system enables genome-wide recovery of newly synthesized RNAs resulting from dynamic TF target interactions. We provide incontrovertible evidence that transiently-bound targets stay in a transcriptionally active state after bZIP1 dissociates from their promoters. The functional read-out captured in our study supports the “hit-and-run” model as a mode of action for a “catalyst” TF to effect genome-wide transcriptional regulation [2, 6, 8]. We propose that this dynamic mode of TF-action enables rapid transcriptional bursting of target genes to enable acute response to external signals. The short exposure time to 4tU used in the *TARGET*-*tU* cell-based system allowed rapid labeling of newly transcribed targets usually overlooked in standard transcriptional studies. Our discoveries of when and how TF-target interactions lead to active transcription, which persists beyond TF-dissociation contributes to the broader field of dynamic transcription networks, with significance beyond plants.

## Methods

### Plant cell preparation and experimental design

Arabidopsis root protoplasts were harvested and transfected as previously described [6, 15]. Briefly, the coding sequence of bZIP1 (At5g49450) was cloned into the destination vector pBeaconRFP_GR. Then, 120 μg of pBeaconRFP_35S::GR::bZIP1 plasmid DNA per 1.5x10^6^ cells was used for protoplast transfection. Cells transfected with the same concentration of empty plasmid pJD385_35S::GR served as a control (Also see Additional file 2: Figure S1). Cells were washed and concentrated by centrifugation, then resuspended in wash solution W5 (154 mM NaCl, 125 mM CaCl2, 5 mM KCl, 5 mM MES, 1 mM Glucose) for overnight incubation at room temperature. Protoplast suspensions were treated sequentially with 20 mM KNO_3_ and 20 mM NH_4_NO_3_ for 2 h, 35 μM cycloheximide (Sigma-Aldrich) for 20 min, and 10 μM dexamethasone (Sigma-Aldrich) for 5 h at room temperature (Fig. 1c). The protoplasts were then treated with 1.5 mM 4tU and 0.2 mM uracil (Fisher). After 20 min of 4tU supply (Additional file 2: Figure S1), protoplasts were FACS sorted to collect 30,000 RFP-positive transfected cells [27] and directly collected into RLT buffer (QIAGEN) for RNA extraction.

### RNA extraction

RNA from 11 replicates (6 biological replicates for bZIP1-transfected cells and 5 replicates for the empty vector) was extracted from protoplasts using an RNeasy Micro Kit with RNase-free DNaseI Set (QIAGEN) and quantified on a Bioanalyzer (Agilent).

### Biotinylation and pull-down of 4tU-labeled fractions

The presence of a thiol group in 4tU-labeled fractions enables conjugation with biotin-HPDP [10]. In detail, total RNA was added to a solution of 10 mM Tris–HCl (Ambion), 1 mM EDTA (Ambion) and 500 ng biotin-HPDP (NEB) and incubated for 3 h in the dark. After centrifugation, the aqueous phase was extracted with an equal volume of chloroform (Sigma-Aldrich) and resuspended in RNase free water.

The biotinylated RNA was fractionated from unlabeled RNA using streptavidin magnetic beads (NEB). Pull-downs were performed as previously described [12]. The beads were washed two times with washing buffer (1 M NaCl, 10 mM EDTA, 100 mM Tris–HCl). The transcripts were then cleaved from magnetic beads using β-mercaptoethanol (5 %, Sigma-Aldrich). The 4tU-labeled fractions were ethanol precipitated and resuspended in RNase free water.

### Microarray hybridization

Pull-down RNA from 4tU-bZIP1 and 4tU-EV fractions was converted into cDNA, amplified and labeled with Ovation Pico WTA System V2 (NuGEN) and Encore Biotin Module (NuGEN), respectively. The labeled cDNA was hybridized, washed and stained on an ATH1-121501 Arabidopsis Genome Array using a Hybridization Control Kit, a GeneChip Hybridization, Wash, and Stain Kit, a GeneChip Fluidics Station 450 and a GeneChip Scanner (Affymetrix).

### Analysis of transcriptomics data

The raw probe intensities were normalized using the GC-robust multiarray averaging package (GCRMA, http://www.bioconductor.org/packages/2.11/bioc/html/gcrma.html). Genes significantly differentially expressed between 4tU-bZIP1 and 4tU-EV fractions were identified as a union of differentially expressed genes determined by either ONE-way ANOVA or Rank Product analysis [28]. The raw p-value of differentially expressed genes was adjusted by False Discovery Rate (FDR) to control for multiple testing. Genes significantly induced and repressed by bZIP1 were then selected with a FDR cutoff of 10 % and only genes with unambiguous microarray probes were kept. The gene dataset was also filtered to account for genes affected by protoplasting and CHX effects, as previously described [6]. Specifically, to avoid any side-effects of CHX, only bZIP1 targets whose DEX-induced expression is the same in either + or – CHX are considered. The heat map in Fig. 2 was created using Multiple Experiment Viewer (Mev) software [29] with expression normalization by z-score for genes/rows to correct the color display between rows. Gene overlaps in Fig. 3 were performed using the Genesect feature of VirtualPlant 1.3 (www.virtualplant.org; [30]) and significance of the overlaps was statistically verified using a hypergeometric test with the microarray as background (the hhyper function in R).

### Gene ontology term enrichment analysis

The set of genes in Fig. 2a and Additional file 4: Figure S2 from repressed and induced bZIP1 targets were analyzed for over-representation of associated GO terms using the Singular Enrichment Analysis feature of agriGO 1.2 [31] using Arabidopsis genome (TAIR10) as a reference and with an FDR cutoff of 5 %.

### *Cis*-element motif analysis

*De novo* motif discovery was performed on 1 kb regions upstream of the transcription start site of the genes actively induced by bZIP1 based on TAIR10 annotation and submitted to the MEME program [32]. Known motif enrichment was performed on 1 kb regions upstream of the transcription start site of the genes actively regulated by bZIP1 based on TAIR10 annotation and submitted to the Elefinder program (http://stan.cropsci.uiuc.edu/tools.php, [33]). The frequency plot of bZIP1-binding motifs in target promoters (Fig. 2b) was generated by plotting the 299 ACGT hybrid boxes identified by Elefinder in the bZIP1 target promoters relative to the distance from the canonical transcription start site (TSS).

### ChIP-Seq data analysis

The chromatin immunoprecipitation sequencing (ChIP-Seq) time-course dataset of bZIP1 binding was previously deposited in the NCBI Sequence Read Archive (SRX425878; [6]). Briefly, Illumina reads were filtered and aligned to the Arabidopsis genome (TAIR10). TF-binding peaks were called using the QuEST package [34] with a ChIP seeding enrichment ≥ 3, and extension and background enrichments ≥ 2. These regions were overlapped with the genome annotation to identify genes within 500 bp downstream of the peak. The profiles of transient bZIP1-targets: *NLP3* (AT4G38340), *THA1* (AT1G08630) and *MCCA* (AT1G03090) in Fig. 4, were shown as bZIP1 ChIP-Seq sequencing depth within 1 kb upstream and downstream the transcription start site.

### ChIP-Seq replication

A second replication of the ChIP-Seq experiment (see Additional file 6: Figure S4) assayed at 5 h time-point after bZIP1 nuclear import was performed following the method described in [6], with the modification that the ChIP and input libraries were sequenced as a fraction of a pool of 12 samples in Illumina MiSeq platform (SE 50 bp).

### Availability of supporting data

The transcriptomics data sets supporting the results of this article are available in the Gene Expression Omnibus [35] repository with accession number GSE69389. The second ChIP-Seq replication performed in this study was deposited to the NCBI Sequence Read Archive with accession number SRR3094677 for ChIP and SRR3095222 for Input. The ChIP-Seq time course and further data used in this analysis were previously deposited [6] and are available in the NCBI Sequence Read Archive with accession: SRX425878.

## Additional files

Additional file 1: Table S1. Comparative tables of the experimental designs (A) and bioinformatics analyses (B) between *TARGET* [6, 15] and *TARGET*-*tU*. (PDF 90 kb) Additional file 2: Figure S1. Experimental design. (A) Vector maps of the pBeaconRFP construct containing 35S::GR::bZIP1 [6, 15] and the pJD385 empty vector (EV) control containing a 35S::GR cassette. (B) A 20 minute exposure to 4tU was sufficient to detect significant incorporation in RNA fractions of Arabidopsis cells, as shown by dot blot imaging of 1 μg RNA from 4.5 10^6^ cells exposed to 1.5 mM 4tU (+) or uracil only (−). (PDF 36 kb) Additional file 3:
**Dataset S1.** Gene lists of bZIP1 targets identified in 4tU-labeled fractions and ChIP-Seq replications. (XLSX 204 kb) Additional file 4: Figure S2. Gene ontology enrichment of bZIP1 targets. GO term enrichment for induced (A) and repressed (B) bZIP1 targets from the 4tU-labeled fractions was performed using agriGO (Toolkit and Database for Agricultural Community, [31]). Significant GO terms were selected from a singular enrichment analysis using Arabidopsis genome (TAIR10) as a reference with an FDR cutoff of 5 %. Significant GO terms levels are highlighted from light yellow to dark red colors. (PDF 2008 kb) Additional file 5: Figure S3 Comparative figure of the mRNA profiles of TF targets assayed by the *TARGET* [6, 15] and *TARGET*-*tU* methodologies. Both methods are comparable and allowed us to provide genome-wide evidence for a “hit-and-run” model of transcription, however the methods can also provide complementary insights based on difference in mRNA populations scored. In detail, *TARGET* [6, 15] measures the steady-state pools of mRNA targets accumulated during the 5 hours time of TF-nuclear localization (mRNA profiles 1 and 2, blue lines), while *TARGET*-*tU* captures mRNA targets actively transcribed when introducing 4-thiouracil (4tU), 5 hours after the TF nuclear import (mRNA profiles 2 and 3, red lines). Using the new *TARGET*-*tU* methodology, we captured a subset of 73 actively transcribed transient targets that overlap with the steady state assays (also see Fig. 3). (PDF 61 kb) Additional file 6: Figure S4. A second ChIP-Seq replication confirms that transient targets are no longer bound by bZIP1 but stay in a transcriptionally active state. A. A comparison of the target overlap identified from the two ChIP-Seq replications performed 5 hours after bZIP1 nuclear import. The first replication of the bZIP1 ChIP-Seq (initially performed in [6]) yielded 850 targets bound by bZIP1 and the second replication (this study) yielded 431 targets bound by bZIP1 (also see Additional file 3: Dataset S1). Here, we obtained 300 common targets between the two replications, this represents a 70 % overlap relative to the replication performed in this study. (B, D) ChIP-Seq binding 1 kb upstream and downstream of the transcription start site (TSS) for 10 additional transient class III targets 5 hours after induced nuclear localization of bZIP1. The stacked plots present the first replication of the bZIP1 ChIP-Seq initially performed in [6] (ChIP-Seq replication 1, top panel) and the second replication performed in in this study (ChIP-Seq replication 2, bottom panel). (C, E) Gene expression levels of 10 additional examples of transient bZIP1 targets in 4tU-labeled fractions of bZIP1 expressing cells (4tU-bZIP1) compared to 4tU-labeled fractions of empty vector (4tU-EV; *FDR < 0.1). Five targets, PIRF1 (AT1G52240), a Dof-type TF (AT1G69570), ALDH6B2 (AT2G14170), ANAC096 (AT5G46590) and ATBXL1 (AT5G49360) are no longer bound by bZIP1 but still show active upregulation (B, C), and five targets, a Chaperone DnaJ-domain superfamily protein (AT1G72070), FRK1 (AT2G19190), RIN4 (AT3G25070), ATHB2 (AT4G16780) and ATAIRP4 (AT5G58787) are no longer bound by bZIP1 but still show active dowregulation (D, E). Please note that the two ChIP-Seq replications (B, D) and *de novo* transcriptomics (C, E) data were performed in independent experiments. (PDF 1244 kb)

## Abbreviations

4tU: 4-thiouracil
bZIP1: basic Leucine Zipper 1
ChIP-Seq: chromatin immunoprecipitation sequencing
EV: empty vector
*TARGET*: Transient Assay Reporting Genome-wide Effects of Transcription factors
TF: transcription factor
