# Supplementary material for: “Hit-and-Run” transcription: de novo transcription initiated by a transient bZIP1 “hit” persists after the “run”
Source: BMC Genomics. 2016 Feb 3;17:92. doi: 10.1186/s12864-016-2410-2 (PMC4738784; doi:10.1186/s12864-016-2410-2)
Supplement: Additional file 1: Table S1. — Comparative tables of the experimental designs (A) and bioinformatics analyses (B) between TARGET [6, 15] and TARGET-tU. (PDF 90 kb) [file 12864_2016_2410_MOESM1_ESM.pdf]

A

	***TARGET* [6, 15]**	***TARGET-tU* (our study)**
**Plant culture**	10 day old Col-0 plants<br>MS medium with KNO_3 1mM<br>Root protoplasting [15, 26]	10 day old Col-0 plants<br>MS medium with KNO_3 1mM<br>Root protoplasting [15, 26]
**Cell treatments and sorting**	Nitrogen, 2h (20mM KNO_3, NH_4NO_3)<br>Cycloheximide (CHX), 20 min<br>Dexamethasone (DEX), 5h<br><br>FACS sorting 10,000 RFP cells	Nitrogen, 2h (20mM KNO_3, NH_4NO_3)<br>Cycloheximide (CHX), 20 min<br>Dexamethasone (DEX), 5h<br>4tU, 20 min<br>FACS sorting 30,000 RFP cells
**RNA extraction**	Total RNA extraction (QIAGEN)	Total RNA extraction (QIAGEN)<br>Pull down of 4tU-labeled fractions [12]
**Microarray**	cDNA synthesis (NuGEN)<br>ATH1-121501 microarray	cDNA synthesis (NuGEN)<br>ATH1-121501 microarray

B

	***TARGET* [6, 15]**	***TARGET-tU* (our study)**
**Normalization**	GCRMA	GCRMA
**Statistical analysis**	ANOVA for bZIP1 effect<br>FDR cutoff 0.05	ANOVA & rank product for bZIP1 effect<br>FDR cutoff 0.1
***cis*-element analysis**	*De novo* motif discovery using<br>MEME software [32]<br>Known motif enrichment using<br>Elefinder software [33]	*De novo* motif discovery using<br>MEME software [32]<br>Known motif enrichment using<br>Elefinder software [33]
**GO terms analysis**	Biomaps feature of VirtualPlant[30]	Singular Enrichment Analysis feature of agriGO [31]
**Gene set overlap**	Genesect feature of VirtualPlant[30]	Genesect feature of VirtualPlant [30]
**Supporting data**	Transcriptome: GSE54049 [6]<br>Chip-Seq: SRX425878 [6]	Transcriptome: GSE69389<br>Chip-Seq: SRX425878 [6]

Additional file 1: Table S1. Comparative tables of the experimental designs (A) and bioinformatics analyses (B) between *TARGET* [6, 15] and *TARGET-tU*.
