# Supplementary material for: “Hit-and-Run” transcription: de novo transcription initiated by a transient bZIP1 “hit” persists after the “run”
Source: BMC Genomics. 2016 Feb 3;17:92. doi: 10.1186/s12864-016-2410-2 (PMC4738784; doi:10.1186/s12864-016-2410-2)
Supplement: Additional file 2: Figure S1. — Experimental design. (A) Vector maps of the pBeaconRFP construct containing 35S::GR::bZIP1 [6, 15] and the pJD385 empty vector (EV) control containing a 35S::GR cassette. (B) A 20 minute exposure to 4tU was sufficient to detect significant incorporation in RNA fractions of Arabidopsis cells, as shown by dot blot imaging of 1 μg RNA from 4.5 106 cells exposed to 1.5 mM 4tU (+) or uracil only (−). (PDF 36 kb) [file 12864_2016_2410_MOESM2_ESM.pdf]

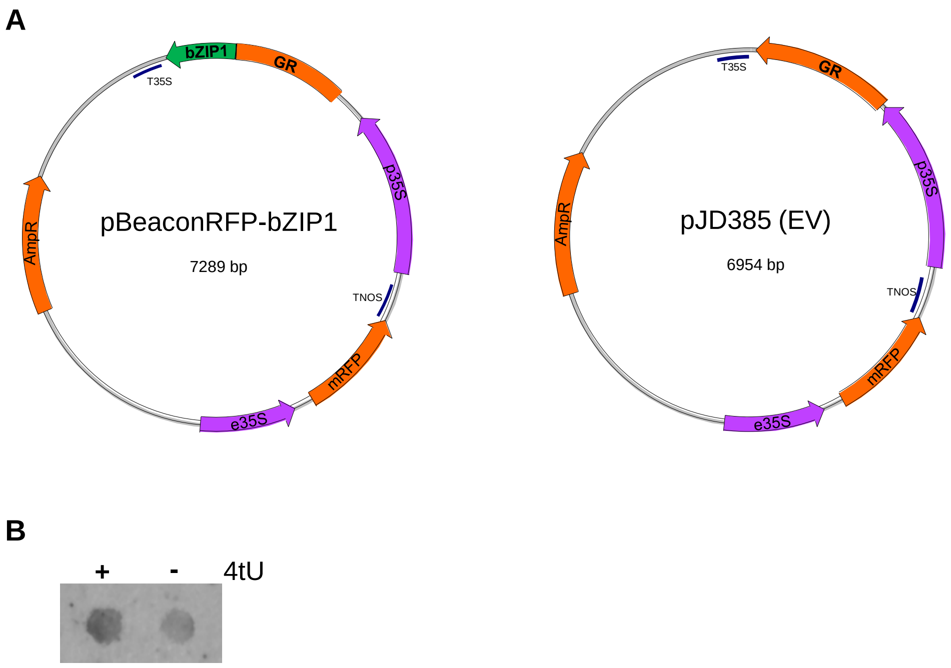

Additional file 2: Figure S1. Experimental design.

(A) Vector maps of the pBeaconRFP construct containing 35S::GR::bZIP1 [6, 15] and the pJD385 empty vector (EV) control containing a 35S::GR cassette. (B) A 20 minute exposure to 4tU was sufficient to detect significant incorporation in RNA fractions of Arabidopsis cells, as shown by dot blot imaging of 1μg RNA from 4.5 10^6 cells exposed to 1.5mM 4tU (+) or uracil only (-).
