# Supplementary material for: “Hit-and-Run” transcription: de novo transcription initiated by a transient bZIP1 “hit” persists after the “run”
Source: BMC Genomics. 2016 Feb 3;17:92. doi: 10.1186/s12864-016-2410-2 (PMC4738784; doi:10.1186/s12864-016-2410-2)
Supplement: Additional file 4: Figure S2. — Gene ontology enrichment of bZIP1 targets. GO term enrichment for induced (A) and repressed (B) bZIP1 targets from the 4tU-labeled fractions was performed using agriGO (Toolkit and Database for Agricultural Community, [31]). Significant GO terms were selected from a singular enrichment analysis using Arabidopsis genome (TAIR10) as a reference with an FDR cutoff of 5 %. Significant GO terms levels are highlighted from light yellow to dark red colors. (PDF 2008 kb) [file 12864_2016_2410_MOESM4_ESM.pdf]

A

B

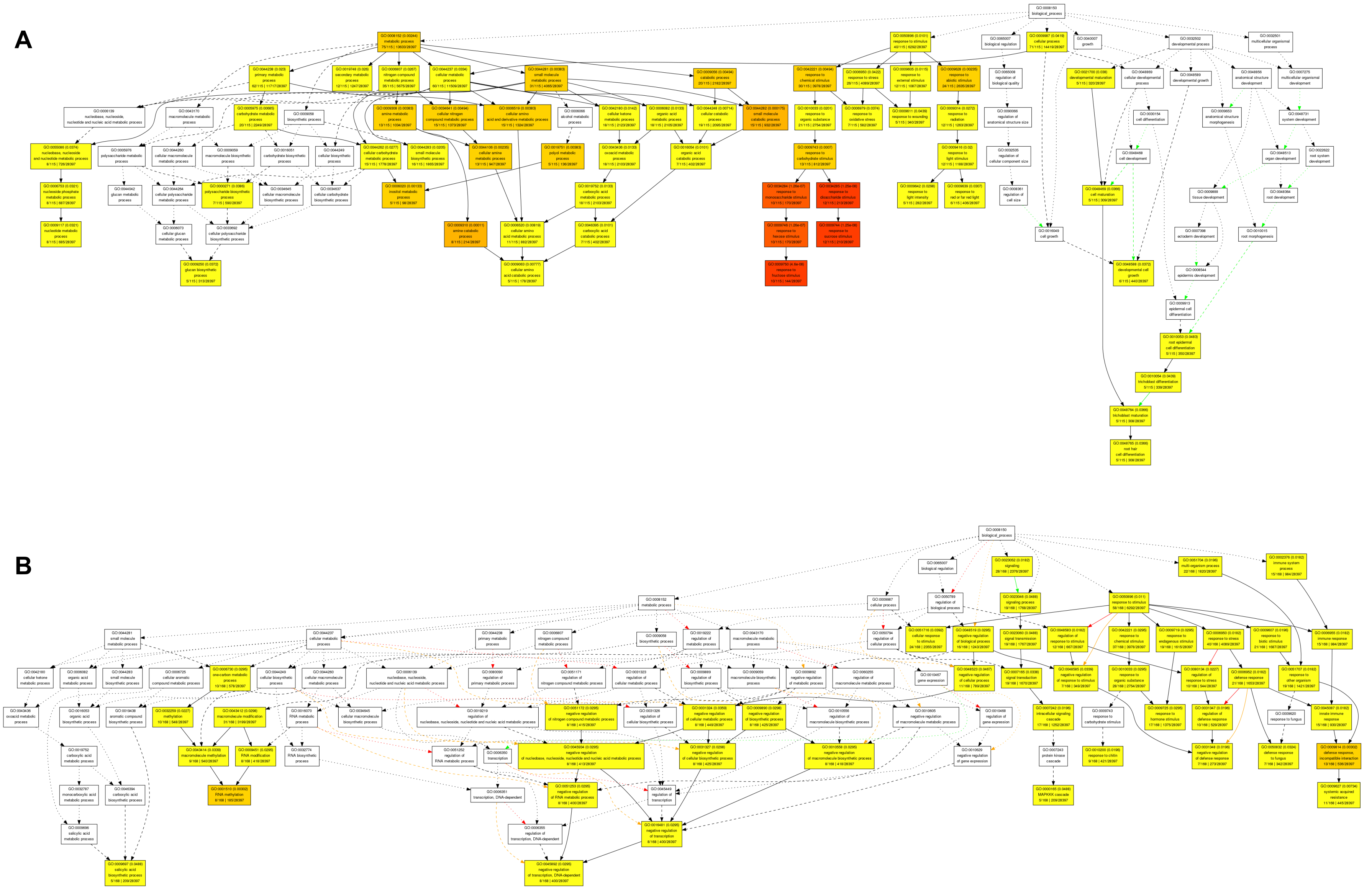

Additional file 4. Figure S2. Gene ontology enrichment of bZIP1 targets.
GO term enrichment for induced (A) and repressed (B) bZIP1 targets from the 4tU-labeled fractions was performed using agriGO (Toolkit and Database for Agricultural Community, [31]). Significant GO terms were selected from a singular enrichment analysis using Arabidopsis genome (TAIR10) as a reference with an FDR cutoff of 5%. Significant GO terms levels are highlighted from light yellow to dark red colors.
