# Supplementary material for: “Hit-and-Run” transcription: de novo transcription initiated by a transient bZIP1 “hit” persists after the “run”
Source: BMC Genomics. 2016 Feb 3;17:92. doi: 10.1186/s12864-016-2410-2 (PMC4738784; doi:10.1186/s12864-016-2410-2)
Supplement: Additional file 5: Figure S3 — Comparative figure of the mRNA profiles of TF targets assayed by the TARGET [6, 15] and TARGET-tU methodologies. Both methods are comparable and allowed us to provide genome-wide evidence for a “hit-and-run” model of transcription, however the methods can also provide complementary insights based on difference in mRNA populations scored. In detail, TARGET [6, 15] measures the steady-state pools of mRNA targets accumulated during the 5 hours time of TF-nuclear localization (mRNA profiles 1 and 2, blue lines), while TARGET-tU captures mRNA targets actively transcribed when introducing 4-thiouracil (4tU), 5 hours after the TF nuclear import (mRNA profiles 2 and 3, red lines). Using the new TARGET-tU methodology, we captured a subset of 73 actively transcribed transient targets that overlap with the steady state assays (also see Fig. 3). (PDF 61 kb) [file 12864_2016_2410_MOESM5_ESM.pdf]

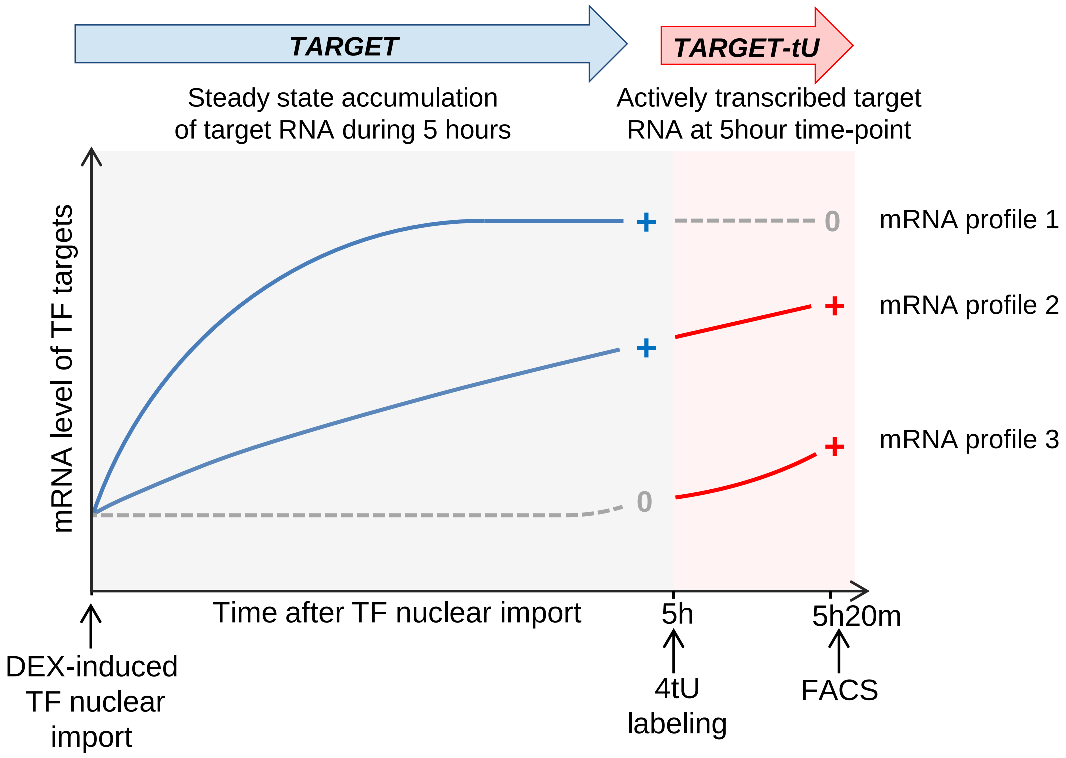

Additional file 5: Figure S3. Comparative figure of the mRNA profiles of TF targets assayed by the *TARGET* [6, 15] and *TARGET-tU* methodologies.

Both methods are comparable and allowed us to provide genome-wide evidence for a "hit-and-run" model of transcription, however the methods can also provide complementary insights based on difference in mRNA populations scored. In detail, *TARGET* [6, 15] measures the steady-state pools of mRNA targets accumulated during the 5 hours time of TF-nuclear localization (mRNA profiles 1 and 2, blue lines), while *TARGET-tU* captures mRNA targets actively transcribed when introducing 4-thiouracil (4tU), 5 hours after the TF nuclear import (mRNA profiles 2 and 3, red lines). Using the new *TARGET-tU* methodology, we captured a subset of 73 actively transcribed transient targets that overlap with the steady state assays (also see Figure 3).
