# Supplementary material for: “Hit-and-Run” transcription: de novo transcription initiated by a transient bZIP1 “hit” persists after the “run”
Source: BMC Genomics. 2016 Feb 3;17:92. doi: 10.1186/s12864-016-2410-2 (PMC4738784; doi:10.1186/s12864-016-2410-2)
Supplement: Additional file 6: Figure S4. — A second ChIP-Seq replication confirms that transient targets are no longer bound by bZIP1 but stay in a transcriptionally active state. A. A comparison of the target overlap identified from the two ChIP-Seq replications performed 5 hours after bZIP1 nuclear import. The first replication of the bZIP1 ChIP-Seq (initially performed in [6]) yielded 850 targets bound by bZIP1 and the second replication (this study) yielded 431 targets bound by bZIP1 (also see Additional file 3: Dataset S1). Here, we obtained 300 common targets between the two replications, this represents a 70 % overlap relative to the replication performed in this study. (B, D) ChIP-Seq binding 1 kb upstream and downstream of the transcription start site (TSS) for 10 additional transient class III targets 5 hours after induced nuclear localization of bZIP1. The stacked plots present the first replication of the bZIP1 ChIP-Seq initially performed in [6] (ChIP-Seq replication 1, top panel) and the second replication performed in in this study (ChIP-Seq replication 2, bottom panel). (C, E) Gene expression levels of 10 additional examples of transient bZIP1 targets in 4tU-labeled fractions of bZIP1 expressing cells (4tU-bZIP1) compared to 4tU-labeled fractions of empty vector (4tU-EV; *FDR < 0.1). Five targets, PIRF1 (AT1G52240), a Dof-type TF (AT1G69570), ALDH6B2 (AT2G14170), ANAC096 (AT5G46590) and ATBXL1 (AT5G49360) are no longer bound by bZIP1 but still show active upregulation (B, C), and five targets, a Chaperone DnaJ-domain superfamily protein (AT1G72070), FRK1 (AT2G19190), RIN4 (AT3G25070), ATHB2 (AT4G16780) and ATAIRP4 (AT5G58787) are no longer bound by bZIP1 but still show active dowregulation (D, E). Please note that the two ChIP-Seq replications (B, D) and de novo transcriptomics (C, E) data were performed in independent experiments. (PDF 1244 kb) [file 12864_2016_2410_MOESM6_ESM.pdf]

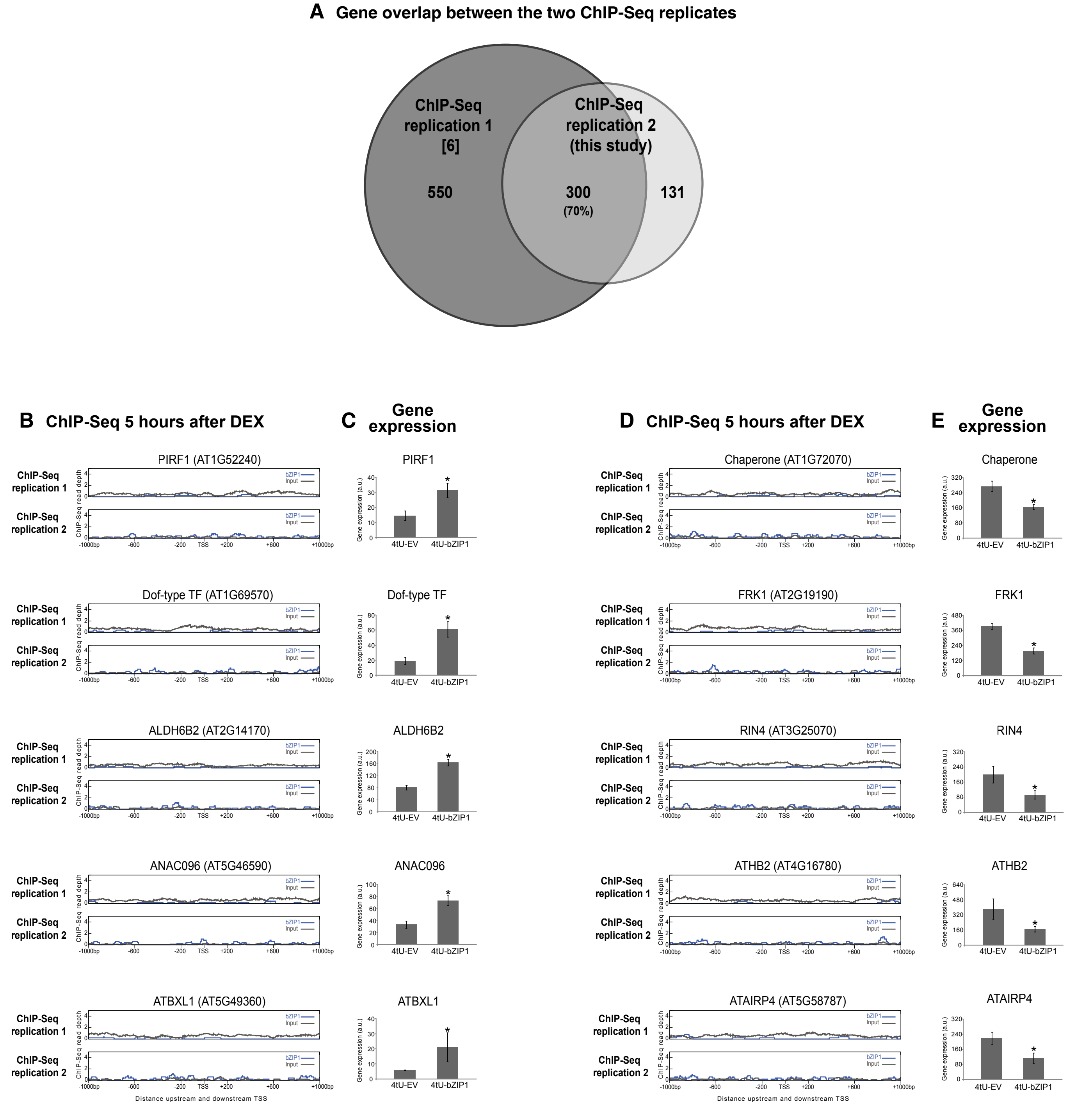

Additional file 6: Figure S4. A second ChIP-Seq replication confirms that transient targets are no longer bound by bZIP1 but stay in a transcriptionally active state.

A. A comparison of the target overlap identified from the two ChIP-Seq replications performed 5 hours after bZIP1 nuclear import. The first replication of the bZIP1 ChIP-Seq (initially performed in [6]) yielded 850 targets bound by bZIP1 and the second replication (this study) yielded 431 targets bound by bZIP1 (also see Additional file 2: Dataset S1). Here, we obtained 300 common targets between the two replications, this represents a 70% overlap relative to the replication performed in this study. (B, D) ChIP-Seq binding 1kb upstream and downstream of the transcription start site (TSS) for 10 additional transient class III targets 5 hours after induced nuclear localization of bZIP1. The stacked plots present the first replication of the bZIP1 ChIP-Seq initially performed in [6] (ChIP-Seq replication 1, top panel) and the second replication performed in in this study (ChIP-Seq replication 2, bottom panel). (C, E) Gene expression levels of 10 additional examples of transient bZIP1 targets in 4tU-labeled fractions of bZIP1 expressing cells (4tU-bZIP1) compared to 4tU-labeled fractions of empty vector (4tU-EV; *FDR<0.1). Five targets, PIRF1 (AT1G52240), a Dof-type TF (AT1G69570), ALDH6B2 (AT2G14170), ANAC096 (AT5G46590) and ATBXL1 (AT5G49360) are no longer bound by bZIP1 but still show active upregulation (B, C), and five targets, a Chaperone DnaJ-domain superfamily protein (AT1G72070), FRK1 (AT2G19190), RIN4 (AT3G25070), ATHB2 (AT4G16780) and ATAIRP4 (AT5G58787) are no longer bound by bZIP1 but still show active dowregulation (D, E). Please note that the two ChIP-Seq replications (B, D) and de novo transcriptomics (C, E) data were performed in independent experiments.
